# Supplementary material for: A Library for fMRI Real-Time Processing Systems in Python (RTPSpy) With Comprehensive Online Noise Reduction, Fast and Accurate Anatomical Image Processing, and Online Processing Simulation
Source: Front Neurosci. 2022 Mar 11;16:834827. doi: 10.3389/fnins.2022.834827 (PMC8963181; doi:10.3389/fnins.2022.834827)
Supplement: Supplementary file 1 [file Data_Sheet_1.pdf]

Supplementary Material

LA-NF application

The scrips in the 'example/LA-NF' directory implement the left amygdala neurofeedback session with happy autobiographical memory recall (1, 2). This script is for a demonstration of a full-fledged GUI application using RTPSpy. Note that this application was not used in the previous studies and several parameters are different from the previous reports. Step-by-step instruction on how to use the application is given on GitHub; https://github.com/mamisaki/RTPSpy/tree/main/example/LA-NF

The neurofeedback run consists of 40s-blocks of Happy, Count, and Rest conditions. At first, the participant is shown the instruction screen (Fig. S1A) that explains each condition. During the Happy condition, the cue 'Happy' and two color bars (red, blue) are displayed on the screen (Fig. S1B). The red bar represents the neurofeedback signal, updated continuously by changing the height based on the neurofeedback signal. The neurofeedback signal is also indicated by a number shown below the red bar. The experiment includes two control conditions ('Count' and 'Rest') to reset the participants' emotional state. During the Count condition (Fig. S1C), a participant is shown the cue 'Count' with the specific instruction to count backward from 300 by subtracting a specified integer. During the Rest condition (Fig. S1D), a participant is presented with the cue 'Rest' and asked to relax while looking at the display screen.

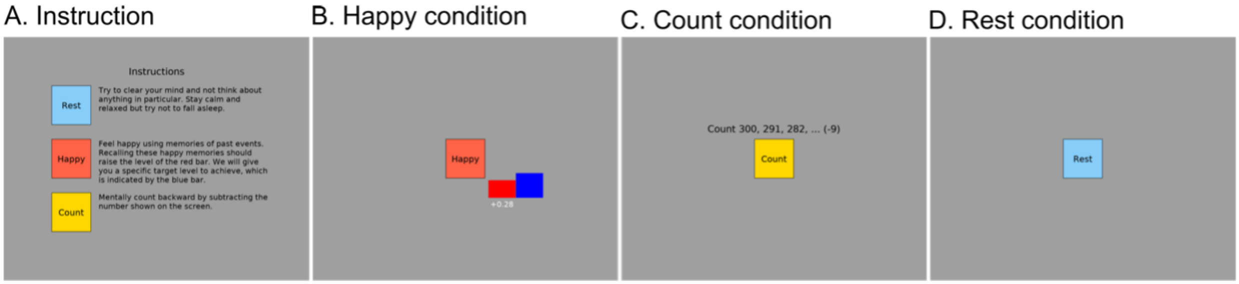

Supplementary figure S1. LA-NF task presentation screens.

The neurofeedback signal is the normalized signal subtracting the mean of the preceding Rest period signal and divided by the standard deviation of the Rest period signal. The Rest period signal used for the normalization is the last 24s signals of the 'Rest' block preceding the Happy block. The neurofeedback signal is computed as a moving average of the current and two available preceding values to reduce bar fluctuations. The initial 4s signals in each Happy block are not presented to wait for the hemodynamic response delay. These calculations are implemented in an external application script, 'example/LA-NF/NF_psypy.py'. RTPSPy application only extracts the mean left amygdala signal from the processed image and sends it to the application.

References

1. Zotev V, Krueger F, Phillips R, Alvarez RP, Simmons WK, Bellgowan P, et al. Self-Regulation of Amygdala Activation Using Real-Time Fmri Neurofeedback. *PLoS ONE* (2011) 6(9):e24522.

2. Young KD, Siegle GJ, Zotev V, Phillips R, Misaki M, Yuan H, et al. Randomized Clinical Trial of Real-Time Fmri Amygdala Neurofeedback for Major Depressive Disorder: Effects on Symptoms and Autobiographical Memory Recall. *Am J Psychiatry* (2017) 174(8):748-55. Epub 20170414. doi: 10.1176/appi.ajp.2017.16060637.
